# Artificial intelligence and robotic technologies redefining precision and personalization in orthopedic surgery: a narrative review

**DOI:** 10.3389/fbioe.2026.1765936

**Published:** 2026-04-14

**Authors:** Fawwaz Al-Smadi, Sajeda Al-Smadi, Xudong Xie, Xiayidan Abudourusuli, Lizhi Ouyang, Ruiyin Zeng, Longyu Du, Yuheng Liao, Bobin Mi, Guohui Liu

**Affiliations:** 1 Department of Orthopedics, Union Hospital, Tongji Medical College, Huazhong University of Science and Technology, Wuhan, China; 2 Pediatric Health Nursing, Department of Allied Medical Sciences, Zarqa University College, Al-Balqa Applied University, Zarqa, Jordan; 3 Department of Public Administration, College of Public Health, Xinjiang Medical University, Urumqi, Xinjiang, China

**Keywords:** artificial intelligence, deep learning, robotic-assisted surgery, orthopedic surgery, preoperative planning, intraoperative navigation, postoperative rehabilitation

## Abstract

The integration of artificial intelligence and robotic technologies is transforming orthopedic surgery by enhancing diagnostic accuracy, surgical precision, and personalized rehabilitation. This review summarizes the development of these technologies, explains their underlying principles, and evaluates their applications across diagnosis, preoperative planning, intraoperative navigation, and postoperative recovery. Artificial intelligence improves early detection of musculoskeletal conditions, supports risk prediction, and enables patient-specific treatment planning through advanced data analysis and image interpretation. Robotic systems enhance surgical consistency and safety by providing real-time guidance, mechanical precision, and controlled execution of complex procedures. Together, these technologies reduce variability, lower complication rates, and support individualized rehabilitation through wearable sensors, motion analysis, and virtual reality platforms. Despite these advances, significant challenges persist, including limited algorithm transparency, data fragmentation, bias, financial barriers, and unresolved ethical and regulatory questions. Future progress is expected to arise from autonomous surgical systems, integration with emerging technologies such as digital twins and high-speed communication networks, and improved data sharing frameworks that support global collaboration. By outlining current capabilities, limitations, and future directions, this review provides a foundation for the responsible and effective adoption of intelligent technologies in orthopedic care.

## 1 Introduction

Artificial intelligence has been increasingly explored across several surgical specialties as a tool for analyzing operative workflows, hand and instrument motion, and technical performance. Recent studies report that AI-based tracking and deep learning-driven assessment methods can be applied to characterize surgical actions and procedural consistency in controlled clinical and experimental settings (Yangi et al., 2025a; Yangi et al., 2025b; Chen et al., 2026). These developments reflect a broader trend toward data-driven surgical practice and provide context for the expanding application of AI within orthopedic surgery.

The field of orthopedic surgery is undergoing a profound transformation as digital technologies increasingly permeate clinical practice (Kurmis and Ianunzio, 2022). Traditionally, the outcomes of orthopedic surgery have depended heavily on the manual dexterity and accumulated experience of the surgeon. However, as the demand for improved clinical outcomes, greater precision, and personalized care intensifies, there is a growing movement toward the integration of artificial intelligence (AI) and robotic-assisted systems into daily practice (Bini, 2018). These technologies promise not only to augment surgical skill but also to enhance diagnostic accuracy, streamline workflows, and reduce variability in patient care.

Although orthopedic surgeons have historically taken pride in the manual expertise of their discipline, the global advancement of medical technologies necessitates a shift in perspective. Early detection and accurate treatment of musculoskeletal disorders continue to pose clinical challenges. For instance, the likelihood of misdiagnosis or underdiagnosis increases by approximately 2.7% when symptoms are vague or nonspecific (Guan et al., 2025). Moreover, it is estimated that 1.1% of fractures are overlooked during initial assessments in emergency departments (Mattijssen-Horstink et al., 2020). These diagnostic shortcomings contribute to increased rates of medical error, delayed interventions, rising treatment costs, and, most importantly, preventable patient harm (Sak and Suchodolska, 2021).

AI encompasses a diverse suite of technologies capable of learning from data, recognizing complex patterns, and making decisions without explicit human programming (Iftikhar et al., 2024). In the medical domain, AI has already demonstrated value across domains such as radiological image analysis (Anyanwu et al., 2025), drug discovery (Paul et al., 2021), and robot-assisted procedures (Knudsen et al., 2024). In orthopedics, clinical algorithms trained on large datasets, including electronic health records, genomic data, imaging studies, and validated scoring tools, are being developed to assist in risk stratification, diagnostic accuracy, and outcome prediction (Lisacek-Kiosoglous et al., 2023). Evidence increasingly supports the role of AI in augmenting clinical reasoning, guiding treatment selection, and enhancing prognostic precision in musculoskeletal care (Lindsey et al., 2018; Sultan et al., 2020; Försch et al., 2021).

Simultaneously, robotic-assisted orthopedic surgery is redefining procedural standards by enabling greater surgical precision and reproducibility (Suarez-Ahedo et al., 2023). The convergence of robotics with AI has further enhanced the capabilities of these platforms, allowing for increased intraoperative efficiency, minimized surgical error, and improved functional recovery (Suarez-Ahedo et al., 2023; Iftikhar et al., 2024). Together, these technologies are now being applied not only in trauma and fracture care but also in complex procedures such as joint arthroplasty and spinal reconstruction, enabling real-time, precision-guided interventions (Rasouli et al., 2021; Hasan et al., 2023).

Despite these advancements, the widespread integration of AI and robotic technologies into daily orthopedic practice remains hindered by several challenges. Data fragmentation, interoperability limitations, and lack of standardized protocols restrict seamless adoption. Moreover, significant legal and ethical concerns, including those surrounding patient data security and clinical accountability, remain unresolved (Altorfer et al., 2025; Cote and Gholipour, 2025; Perez and Garcia, 2025).

Although numerous reviews have examined artificial intelligence or robotic-assisted technologies in orthopedic surgery, most have focused on AI-based decision support systems or robotic platforms in isolation. Existing literature often focuses on specific subspecialties, individual algorithms, or particular robotic platforms rather than examining how AI and robotics jointly interact within integrated orthopedic workflows spanning diagnosis, surgical planning, intraoperative assistance, and postoperative rehabilitation. Recent evidence syntheses have also highlighted the rapidly expanding body of research on artificial intelligence applications in orthopedic surgery (Geda et al., 2024; Han et al., 2025). However, the interaction between AI and robotics within integrated orthopedic workflows across the diagnostic, surgical, and rehabilitative continuum remains insufficiently explored.

**FIGURE 1:**
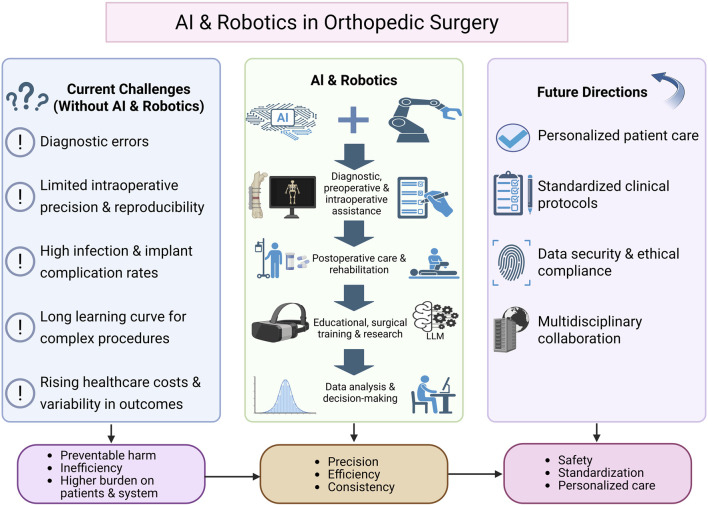
Conceptual schematic summarizing major challenges in orthopedic care and the roles of AI and robotic technologies across diagnosis, preoperative planning, intraoperative assistance, postoperative care, education, and data-driven decision-making. This figure is a conceptual schematic drawn based on evidence and references discussed throughout Sections 1–6 of the manuscript.

In the context of rapid technological development and fragmented clinical adoption, there is an urgent need for a narrative review that consolidates current evidence, delineates enabling technologies, and charts a course for future development. Therefore, this review aims to provide an overview of the integration of AI and robotics in orthopedic surgery—examining current applications, potential value, and critical challenges. In doing so, we seek to establish a scientific foundation and strategic roadmap for the safe, standardized, and personalized implementation of these technologies. We hope this review will serve as a valuable decision-making framework for clinicians, while also guiding researchers and developers toward focused innovation and meaningful collaboration in the advancement of AI-driven orthopedic care. A schematic overview summarizing these challenges, the role of AI and robotics, and potential future directions in orthopedic surgery is presented in Figure 1.

## 2 Literature search strategy

This narrative review was informed by a literature search to identify recent and relevant studies on artificial intelligence and robotic technologies in orthopedic surgery. Searches were conducted in PubMed, Web of Science, Scopus, and Google Scholar for articles published up to November 2025. Search terms included combinations of the following keywords: “artificial intelligence,” “machine learning,” “deep learning,” “robotic-assisted surgery,” “orthopedic surgery,” “arthroplasty,” “spine surgery,” “trauma,” “rehabilitation,” and “computer vision.”

The representative search syntax used in PubMed was: (“artificial intelligence” OR “machine learning” OR “deep learning” OR “computer vision”) AND (“orthopedic surgery” OR orthopaedic OR arthroplasty OR “spine surgery” OR trauma OR fracture OR rehabilitation) AND (robotic* OR “robot-assisted” OR “robotic-assisted”).

Peer-reviewed, English-language articles describing clinical applications, technical developments, validation studies, and relevant narrative or systematic reviews were considered for inclusion. Studies focusing on orthopedic surgery were prioritized; however, high-quality articles addressing general surgical applications of artificial intelligence and robotic technologies were also included when they provided important contextual or methodological insights relevant to orthopedics. Non-peer-reviewed opinion articles were excluded. Reference lists of key articles were manually screened to ensure inclusion of seminal and recent publications.

Article selection and thematic synthesis were performed independently by two authors. Discrepancies were resolved through discussion with a third experienced author to reach consensus. Emphasis was placed on clinical relevance and contribution to understanding AI- and robotics-enabled orthopedic workflows. As this work was designed as a narrative review, formal PRISMA-based screening procedures, record counts, and risk-of-bias assessments were not performed. However, the search process was conducted in a structured manner to ensure transparency and comprehensive coverage of relevant literature.

## 3 Foundations and integration of artificial intelligence with robotic systems

### 3.1 History of AI and robotic technologies

Artificial intelligence and robotic technologies emerged from distinct technological trajectories but have increasingly converged in modern medicine, particularly in orthopedic surgery. The conceptual foundations of AI date back to mid-twentieth-century computational theory, with early developments in machine intelligence and decision-making frameworks following the pioneering work of Alan Turing and the formal introduction of the term “artificial intelligence” at the Dartmouth Conference in 1956 (Fontana et al., 2019; Savadjiev et al., 2020; Karnuta et al., 2021). Over the following decades, AI evolved from rule-based expert systems to data-driven machine learning models and, more recently, to deep learning architectures capable of extracting complex patterns from large-scale datasets (Yin et al., 2022; Fritz et al., 2023). Breakthroughs such as generative adversarial networks (GANs) (Yi et al., 2019; Li, 2022; Serrano, 2023) and convolutional neural networks (CNNs) (Litjens et al., 2017; Balaji et al., 2024; Wang H. et al., 2024) enabled machines to extract complex features directly from raw data and generate realistic synthetic outputs, expanding the scope of medical applications (LeCun et al., 2015).

Parallel to the development of AI, robotic surgery has undergone a similarly transformative trajectory. The first clinically relevant robotic-assisted procedures date back to the 1980s with the introduction of the PUMA 560 robotic arm, which was initially employed for stereotactic neurosurgery (Theodore et al., 2018). The U.S. FDA’s approval of the da Vinci Surgical System in 2000 represented a landmark moment, signifying the transition of robotic surgery from experimental to mainstream clinical use (Iftikhar et al., 2024). In orthopedics, robotic technologies began to gain prominence in the early 1990s with the introduction of the ROBODOC system for total hip arthroplasty (THA), representing one of the earliest applications of robotic assistance in orthopedic procedures. Since then, a wide array of robotic platforms has been developed for high-precision interventions in spine, knee, and trauma surgery, reflecting a growing demand for reproducible outcomes and minimally invasive techniques (Shah et al., 2014).

**FIGURE 2:**
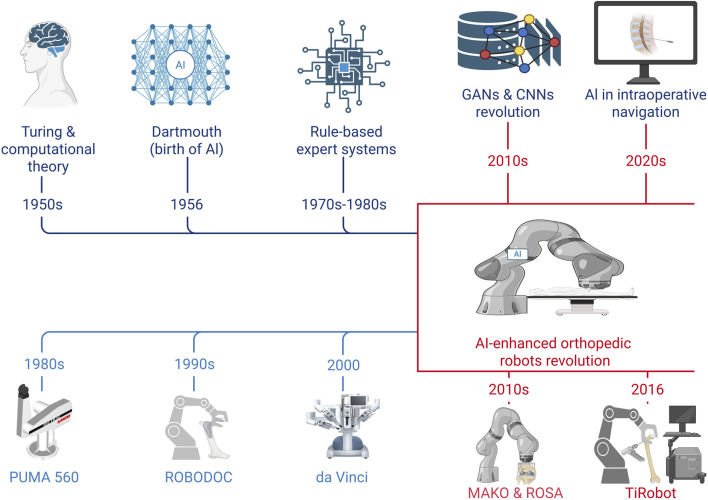
Schematic timeline of key milestones in the development and convergence of artificial intelligence and robotic surgery in medicine. This figure is a conceptual schematic drawn based on evidence and references discussed in Section 3.1.

More recently, the convergence of AI and robotics has enabled the development of intelligent surgical systems capable of integrating advanced imaging, data analytics, and real-time intraoperative guidance. These systems allow automated identification of anatomical structures, enhanced surgical planning, and improved procedural precision, contributing to safer and more efficient orthopedic interventions (Pierson and Gashler, 2017; Zheng et al., 2021; Lee R. Y. et al., 2023). A timeline of key technological milestones in the evolution and convergence of AI and robotics in medicine is illustrated in Figure 2.

### 3.2 Artificial intelligence technologies

AI in orthopedic surgery encompasses a broad and rapidly evolving range of technologies, including deep learning (DL), machine learning (ML), artificial neural networks (ANNs), natural language processing (NLP), reinforcement learning (RL), and computer vision (CV) (Fonseca et al., 2025). ML, a foundational subset of AI introduced by Arthur Samuel in 1959 (Dowell et al., 2023), involves algorithmic systems that autonomously learn from data patterns without explicit programming. These models, trained on large and often heterogeneous datasets, can identify latent structures, predict outcomes, and generalize to novel clinical scenarios (Jordan and Mitchell, 2015). However, traditional ML often requires feature engineering and supervision.

NLP enables computers to interpret, analyze, and manage human language in textual form (Myers et al., 2020). In orthopedic practice, it is particularly valuable for extracting clinically relevant information from unstructured sources such as surgical reports and radiology notes. These capabilities enable the automation of data collection, potentially enhancing diagnostic accuracy and improving patient care workflows (Fu et al., 2021). NLP may also support the aggregation and analysis of large-scale databases that are typically infeasible to curate manually, thereby reducing clinician workload and enabling real-time cohort tracking (Myers et al., 2020; Wyatt et al., 2021). Practical applications include identifying periprosthetic joint infections and organizing records related to periprosthetic fractures (Fu et al., 2021; Yi et al., 2022).

RL has gained prominence as a flexible AI paradigm for managing sequential decision-making in healthcare (Ali, 2022). In contrast to supervised learning approaches that rely on labeled data, RL systems learn through interactions with their environment, guided by reward-penalty feedback loops (Ermolieva et al., 2021). RL algorithms offer a data-driven alternative to human judgment by continuously updating based on real-time performance, historical outcomes, and evolving clinical contexts. In orthopedics, RL can dynamically adjust surgical strategies, allocate resources, and personalize treatment regimens, making it highly relevant to precision medicine initiatives (Aldahiri et al., 2021; Ali, 2022). Its suitability for handling uncertain and complex clinical environments underpins its growing role in surgical training, robotic navigation, and outcome optimization.

Computer vision enables machines to interpret and analyze medical images using AI-driven neural networks (Prijs et al., 2022). In orthopedic contexts, CV systems have been developed for fracture detection and classification across modalities such as radiographs and computed tomography (CT) imaging (Langerhuizen et al., 2019; Carmo et al., 2021). By processing visual inputs, often in standardized formats like Digital Imaging and Communications in Medicine (DICOM), these systems can achieve diagnostic performance comparable to, or even exceeding, that of clinicians. However, challenges persist in validating their outputs and ensuring reliability in clinical decision-making, which remains a major barrier to widespread adoption (Prijs et al., 2022).

DL represents a more advanced and scalable subset of ML, characterized by multilayered neural network architectures such as ANNs that are capable of modeling complex nonlinear relationships (Beyaz et al., 2020; Purnomo et al., 2021; Farhadi et al., 2022). Unlike conventional ML models, DL models typically operate with billions of parameters, enabling them to perform high-dimensional data processing with minimal manual intervention. Although many DL applications still depend on supervised learning, there is growing interest in self-supervised and unsupervised approaches that leverage unstructured and unlabeled datasets. In orthopedic surgery, CNNs, a specialized class of DL models, are extensively used for medical image analysis, including lesion detection, classification, and segmentation (Yamashita et al., 2018; Purnomo et al., 2021).

CNNs are a class of neural architectures that apply convolutional operations across layers to identify spatial features and patterns in data (Derry et al., 2023). Their ability to learn feature hierarchies makes them particularly effective for tasks such as visual recognition and image-based diagnosis. These models are widely adopted in medical AI, particularly for computer vision and NLP tasks (Guan et al., 2025). Within the medical domain, and particularly in orthopedics, CNNs have become the leading model for tasks involving disease detection, diagnosis, and therapeutic planning (Derry et al., 2023). Current applications include tumor identification, image classification, and segmentation, underscoring the increasing relevance of CNNs in orthopedic diagnostic workflows (Lisacek-Kiosoglous et al., 2023).

The design and development of AI algorithms tailored for orthopedic surgery must accommodate the complexity and heterogeneity of clinical data. Conventional statistical and machine learning approaches often face difficulties in integrating multiple data modalities, limiting their applicability in high-dimensional clinical environments. In contrast, modern AI systems facilitate ongoing algorithmic refinement, multimodal fusion, and scalable learning frameworks (He et al., 2025). A significant barrier in orthopedic AI development lies in the variety of data types, ranging from preoperative imaging to intraoperative physiological metrics and postoperative functional measures, each differing in structure, resolution, and temporal granularity (Han et al., 2025). Effective integration of such diverse datasets through robust algorithmic frameworks enhances model accuracy and clinical relevance, thereby improving clinical decision-making, surgical precision, and postoperative care (Zsidai et al., 2023; Bozzo et al., 2024; Chakraborty et al., 2025). A summary of commonly employed and emerging AI algorithms in orthopedic surgery, together with representative task domains (e.g., imaging-based diagnosis, preoperative planning, intraoperative navigation, and rehabilitation monitoring), is presented in Table 1, with detailed clinical applications discussed in Section 5.

**TABLE 1:** Contemporary AI algorithms in orthopedic surgery: clinical applications, strengths, and limitations.

Algorithm type	Application scenarios	Advantages	Limitations
Supervised ML Models (Wirries et al., 2022; Arad et al., 2023; Pruneski et al., 2023a)	Postoperative outcome prediction, implant durability	High prediction accuracy; improves individualized care	Limited interpretability; sensitive to missing clinical data
NLP (Pruneski et al., 2023b; AlShehri et al., 2024; Morya et al., 2024)	Clinical documentation analysis, surgical planning	Automates data extraction from Electronic Health Records; enhances report generation	Performance varies with language structure; limited multilingual capability
RL (Ovinnikov et al., 2024; Qian and Ren, 2025)	Robotic path optimization, surgical simulation	Adapts to dynamic intraoperative changes; improves precision	Complex training process; requires real-time feedback data
Decision trees and ensemble learning (Valliani et al., 2022)	Fracture staging, metastasis detection, risk prediction	Effective with small datasets; interpretable results	Susceptible to overfitting; dependent on feature selection
3D modeling and simulation algorithms (Shi et al., 2022; Mendonça et al., 2023)	Preoperative planning, prosthesis design	Enables high-precision visualization; supports surgical education	Labor-intensive modeling; image quality-dependent
CNNs (Liao et al., 2022)	Image segmentation, disease identification	Captures hierarchical features across tissues (e.g., cartilage, bone); reduces diagnostic subjectivity	High computational cost; needs high-resolution input
DL (Yang et al., 2022; Yao et al., 2025)	Fracture classification, bone tumor detection	Integrates multi-dimensional imaging data; enables automated surgical planning	Requires extensive annotated datasets; generalization may be limited
Real-time navigation algorithms (Lu et al., 2022; Li et al., 2025)	Robotic-assisted procedures	Enhances surgical accuracy; reduces radiation exposure	High operational cost; requires specialized infrastructure
Automated osteotomy control algorithms (Przystalski et al., 2023; Hodel et al., 2024; Mitterer et al., 2024)	Joint arthroplasty robotics	High-precision cutting; minimizes surgeon variability	Relies on CT-based planning; costly setup
Image segmentation algorithms (e.g., U-Net) (Song et al., 2023; Wang et al., 2023b; Ling et al., 2024)	Precise tissue delineation	Assists navigation; improves intraoperative targeting	Requires annotated data; high computational load
Object detection algorithms (YOLO/SSD) (Doerrich et al., 2023; Fahlbusch et al., 2024; Han et al., 2024)	Pre/intraoperative lesion detection	Enables real-time guidance; enhances diagnostic workflow	Less effective for small targets; hardware-intensive
XGBoost (Jiang et al., 2024a; Liu et al., 2024; Zhang et al., 2024a; Wu et al., 2025)	Postoperative complication detection	Performs well on small, structured datasets; interpretable results	Manual feature engineering required; sensitive to noisy inputs

### 3.3 Integration of AI with robotic systems

The convergence of AI with robotic systems is rapidly transforming orthopedic surgery. Although robots and AI belong to different technical categories—the former focuses on mechanical execution and precision control, and the latter on data analysis, prediction, and decision support—their integration in orthopedics is becoming increasingly close. Advances in AI, particularly in natural language processing, medical imaging, and clinical decision support, have expanded the capabilities of robotics beyond mechanical execution to include personalized planning and intelligent intraoperative navigation. These integrated systems are now routinely applied across orthopedic subspecialties to enhance surgical precision and outcomes.

In total knee arthroplasty (TKA), robot-assisted procedures have been reported to demonstrate improved implant alignment and bone resection accuracy when compared to conventional approaches (Jacofsky and Allen, 2016; Troccaz et al., 2019). Similar advantages are observed in THA, where robotic assistance enables more precise acetabular and femoral component placement, thereby improving joint stability, reducing leg length discrepancies, and preserving bone stock (Jacofsky and Allen, 2016; Kim et al., 2023). In spinal surgery, robotic navigation improves pedicle screw placement accuracy, thereby reducing neurological complications (Ahern et al., 2020). For trauma cases, robotic systems facilitate accurate screw trajectory and minimize intraoperative errors, contributing to safer, more efficient procedures (Zheng et al., 2021).

The contribution of AI extends these capabilities beyond physical execution. Robotic platforms deliver high-fidelity mechanical execution, whereas AI contributes predictive modeling, intraoperative decision support, and individualized surgical planning (Farhadi et al., 2022). Evidence indicates that AI-assisted preoperative planning can reduce the need for intraoperative modifications by 39.7% in selected cohorts compared with conventional approaches, highlighting the efficiency of algorithm-driven workflows (Lambrechts et al., 2022). Beyond preoperative optimization, AI also facilitates real-time intraoperative guidance, enabling adaptive responses to surgical complexity (Lambrechts et al., 2022). A notable example is the TiRobot system, which integrates a robotic arm with real-time navigation to support precise interventions in spinal, pelvic, and femoral neck procedures (WANG et al., 2021b; Zhu et al., 2021; Al-Smadi et al., 2025).

**FIGURE 3:**
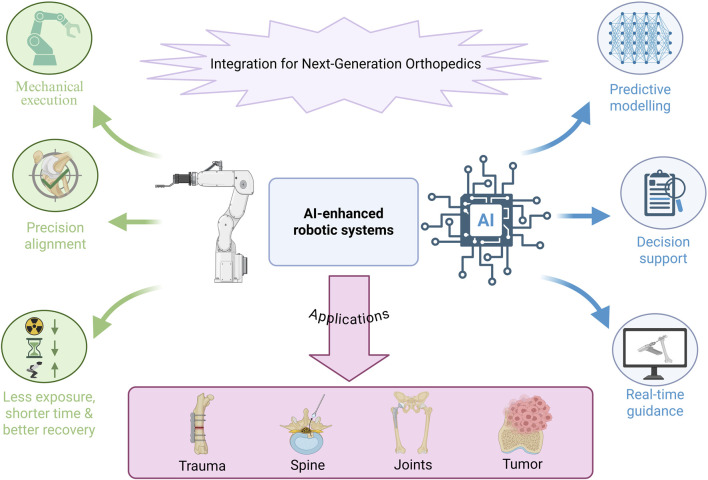
Conceptual illustration demonstrating how AI-enabled decision support interfaces with robotic-assisted execution to enhance surgical precision, efficiency, and consistency across orthopedic procedures. This figure is a conceptual schematic drawn based on evidence and references discussed in Sections 3.3, 3.4.

Beyond improving precision, robotic systems integrated with computer-assisted navigation have been shown to reduce operative duration, anesthesia exposure, and radiation risk. These platforms decrease reliance on fluoroscopy by offering enhanced anatomical visualization, thereby improving both surgeon safety and workflow efficiency (Overley et al., 2017; Kochanski et al., 2019; Wang et al., 2021b; Zhu et al., 2021; Kim et al., 2023; Massé et al., 2023). Clinical outcomes are likewise encouraging; robotic procedures have been associated with higher scores in patient-reported metrics, including the Forgotten Joint Score (FJS) and Harris Hip Score (HHS) (Overley et al., 2017; Kochanski et al., 2019; Domb et al., 2020; Wang et al., 2021b; Zhu et al., 2021; Kim et al., 2023; Massé et al., 2023). These applications and benefits are summarized in Figure 3.

### 3.4 Classification of robotic systems in orthopedics

Within the framework of AI-robotics integration, orthopedic robotic systems can be categorized using multiple criteria, including platform type, method of anatomical data acquisition, and control architecture (Innocenti and Bori, 2021). From a platform perspective, open systems, exemplified by the TMINI platform (formerly ROBODOC; THINK Surgical, Fremont, CA), are compatible with prosthetic implants from a range of manufacturers, offering surgeons greater flexibility in implant choice (Ponna et al., 2025). By contrast, closed systems, such as Mako SmartRobotics (Stryker, Kalamazoo, MI), ROSA Knee (Zimmer Biomet, Warsaw, IN), CORI (Smith and Nephew, London, United Kingdom), and Apollo (Corin, Cirencester, United Kingdom), are engineered to work exclusively with manufacturer-specific implants, enabling streamlined integration but limiting implant selection (Innocenti and Bori, 2021; Ponna et al., 2025). Although open platforms promote adaptability, certain models operate without patient-specific imaging, relying instead on generalized anatomical landmarks, which may compromise accuracy in anatomically complex scenarios (Ponna et al., 2025).

A second axis of classification relates to how anatomical data are acquired. Image-based systems employ preoperative imaging, such as magnetic resonance imaging (MRI) or CT, to generate high-resolution three-dimensional anatomical reconstructions for surgical planning. Examples include TMINI, Mako, and ROSA, which provide excellent accuracy but incur additional costs and may expose patients to extra radiation (Innocenti and Bori, 2021; Ponna et al., 2025). In contrast, imageless systems such as Apollo and CORI capture anatomical information intraoperatively through surface mapping and registration (Innocenti and Bori, 2021). While these systems reduce preoperative requirements and eliminate radiation exposure, their precision is heavily dependent on the surgeon’s ability to capture accurate intraoperative landmarks (Innocenti and Bori, 2021).

At the human-machine interaction level, active systems, such as the early-generation ROBODOC and CASPAR (Ortho-Maquet/URS), carry out pre-programmed tasks—such as bone resection—with minimal surgeon input (Ponna et al., 2025). Although capable of high precision, these systems have been linked to prolonged operative times and complications, including iatrogenic fractures and ligament injuries, due to limited intraoperative adaptability (Siebel and Käfer, 2005; Innocenti and Bori, 2021). Passive systems, by contrast, perform no autonomous surgical actions; instead, they provide real-time guidance while the surgeon maintains complete control. The Apollo platform exemplifies this model, enhancing accuracy via adjustable cutting guides and feedback mechanisms, without robotic actuation (Innocenti and Bori, 2021). Semi-active systems combine elements of both approaches, enabling manual instrument control while the robot enforces pre-defined safety boundaries through haptic feedback or motion constraints. Platforms such as Mako and CORI employ this configuration to maintain surgical precision and adaptability (Innocenti and Bori, 2021). Similarly, the TiRobot system (TINAVI) integrates AI-assisted preoperative planning with real-time navigational guidance for orthopedic trauma procedures involving the spine, pelvis, and femur, while ensuring the surgeon retains authority over all critical operative steps (Wang et al., 2021b; Zhu et al., 2021; Al-Smadi et al., 2025).

To more intuitively present the technical characteristics and differences of various orthopedic robotic systems in terms of platform structure, image dependence, control mode, AI fusion path, and clinical applicability, representative platforms are summarized in Table 2. Because these commercial robotic systems have been evaluated across numerous clinical studies and surgical cohorts, the table focuses on technological characteristics and clinical functionality rather than reporting validation sample sizes from individual studies.

**TABLE 2:** Summary of orthopedic surgical robotic systems: core technologies, AI integration, applications, and limitations.

Robotic system	Technology	AI integration level	Applicable surgical procedures	Advantages	Limitations
ROBODOC/TMINI (Think Surgical) (Paul et al., 1992; Siebert et al., 2002)	Fully active robotic arm for preoperative CT-based bone milling	None	Total hip arthroplasty	High milling precision; excellent fit for implants	Pre-defined, static; lacks intraoperative adaptability
Mako (Stryker) (Kayani et al., 2019; Roche, 2021; Nam et al., 2022)	Semi-active robotic arm with CT-based planning and haptic feedback	Rule-based system	Total hip, total knee, partial knee arthroplasty	Haptic feedback for surgeon; precise bone resection	Requires pre-op CT; limited intraoperative adaptability
NAVIO (Smith and Nephew) (Battenberg et al., 2020; Bell et al., 2022)	Semi-active robot with real-time intraoperative mapping, imageless	Rule-based system	Total knee, partial knee arthroplasty	CT-free workflow; flexibility; faster intraoperative adjustments	Requires extensive intraoperative registration and clamping
TiRobot (TINAVI) (WANG et al., 2021b; Zhu et al., 2021; Al-Smadi et al., 2025)	Robotic arm with imaging-based navigation (X-ray/CT/MRI)	Data-driven/AI-assisted	Spine, pelvis, femoral neck fractures	High accuracy; complex anatomy navigation; reduced radiation exposure to surgeon and patient	Scew pathway predefined by surgeon; lacks intraoperative adaptive learning
ROSA (Zimmer Biomet) (Batailler et al., 2021; Siddiqi et al., 2021)	Semi-active system with 2D-3D integration and intraoperative analytics	Data-driven/AI-assisted	Total knee arthroplasty	Dynamic 3D planning; intraoperative adjustment; improves implant positioning	Requires additional intraoperative hardware and clamping
ExcelsiusGPS (Globus Medical) (Jiang et al., 2018; Huang et al., 2021)	Robotic guidance for spine screws with integrated navigation	Rule-based system	Spine surgery	High precision in complex spine anatomy; real-time correction; radiation safety	No haptic feedback; surgeon relies on visual guidance
CORI (Smith and Nephew) (Adamska et al., 2023; Weaver et al., 2024)	Portable system with real-time mapping (evolved from NAVIO)	Rule-based system	Total knee, partial knee arthroplasty	Compact, portable; no pre-op CT; efficient workflow	Long learning curve; relies on intraoperative surgeon skill

### 3.5 Challenges facing AI-Integrated robotics systems

Although AI-enabled robotic systems have demonstrated substantial clinical benefits in orthopedic surgery—enhancing surgical precision, lowering complication rates, and advancing individualized decision-making—their large-scale clinical deployment continues to face multiple challenges. These challenges are primarily concentrated in the domains of technology dissemination, economic accessibility, organizational implementation, and regulatory oversight.

From an economic perspective, the high initial procurement costs, together with ongoing expenses for system maintenance, technical support, and consumables, remain among the most significant barriers to widespread adoption. The return on investment is often protracted in small- to medium-sized hospitals and in primary or rural healthcare settings, where limited resources and lower patient volumes reduce utilization rates, thereby exacerbating disparities in access to advanced surgical technologies across regions (St Mart and Goh, 2021; Kolessar et al., 2022; Peterman et al., 2024). Against this backdrop, optimizing cost structures and facilitating the diffusion of AI-assisted robotic systems, particularly in low- and middle-income countries and resource-limited settings, has become a critical component of the global agenda for equitable surgical innovation.

Another barrier is the pronounced learning curve, which requires surgical teams to undergo structured training, adapt to new equipment and role assignments, and acquire higher levels of technical proficiency. While mentorship from experienced robotic surgeons can mitigate adaptation challenges, comprehensive training and credentialing remain essential for ensuring both safety and clinical efficacy (Ali et al., 2022; Schopper et al., 2023). For surgeons transitioning from conventional to robot-assisted techniques, the initial phase often involves longer operative times, which generally improve with growing familiarity and workflow optimization (Sodhi et al., 2018; Chen et al., 2023). Furthermore, although hardware and software failures are uncommon, their occurrence can lead to intraoperative delays or necessitate conversion to conventional methods, with potential implications for surgical outcomes (Nogalo et al., 2023).

At the regulatory level, AI-driven surgical systems, owing to their algorithmic complexity, real-time decision-making, and varying degrees of autonomy, face lengthy and stringent approval processes, which can delay product deployment and scalability (Marrone et al., 2022; Yi, 2024). Their integration into robotic surgical workflows also demands a fundamental reconfiguration of procedure design, intraoperative navigation, and postoperative assessment protocols. Consequently, all members of the perioperative team, including surgeons, nurses, and technical staff, must receive formal training and certification to ensure safe, effective, and standardized use of these systems in clinical settings (Romagna et al., 2023; Zhou et al., 2025).

Equally important are the escalating concerns over cybersecurity and data privacy as AI systems become deeply embedded in surgical care. Potential risks—such as system hacking, algorithmic malfunction, or compromised remote control—pose direct threats to patient safety and surgical outcomes (Naik et al., 2022; Thorn, 2023). To address these challenges, regulatory authorities should establish unified, transparent, and continuously updated approval and safety frameworks, while technology providers must implement robust cybersecurity and encryption protocols. These measures are essential for safeguarding operational stability, protecting patient rights, and fostering public trust in intelligent robotic surgical technologies.

## 4 Applications of AI and robotic technologies in orthopedic surgery

Artificial intelligence applications in orthopedic surgery span multiple stages of clinical care, including diagnosis, preoperative planning, intraoperative navigation, and postoperative rehabilitation. While numerous studies have explored these applications, the methodological characteristics of representative investigations are summarized in Table 3 to provide context regarding study design, sample size, validation approaches, and reported outcomes.

**TABLE 3:** Representative studies evaluating AI applications across major orthopedic domains.

Domain	Study	AI/Robotic technology	Study design	Sample size	Validation	Primary outcome	Key finding
Diagnosis and Imaging	Olczak et al. (2017)	CNN	Retrospective imaging study	256,000 radiographs	Train/validation/test split	Fracture detection accuracy	83% fracture detection accuracy comparable to orthopedic surgeons
Diagnosis and Imaging	Lindsey et al. (2018)	Deep CNN	Deep learning diagnostic study with clinician experiment	135,845 radiographs + 40 clinicians	External test datasets + clinician experiment	Diagnostic sensitivity improvement	Clinician sensitivity improved from 80.8% to 91.5%; misinterpretation reduced by 47%
Diagnosis and Imaging	Inoue et al. (2022)	CNN	Retrospective imaging study	200 patients, 7,664 CT slices	Train/validation/test split	Vertebral fracture detection	Sensitivity 0.786; improved fracture detection with AI assistance
Preoperative planning	Karnuta et al. (2020)	ANN	Retrospective ML cohort study	111,147 shoulder arthroplasty patients	Train/validation/test split	Hospital LOS, cost, discharge prediction	LOS prediction accuracy 91.8%, discharge prediction 73.1%
Preoperative planning	Wei et al. (2021)	ANN	Retrospective cohort (NSQIP database)	28,742 TKA patients	Internal validation vs. logistic regression	Same-day discharge prediction	ANN achieved AUC 0.801 for predicting outpatient TKA discharge
Intraoperative Assistance	Zhu et al. (2021)	TiRobot navigation	Retrospective comparative clinical study	133 patients (50 TiRobot vs. 83 conventional)	Single-center clinical comparison	Screw placement accuracy	Improved screw placement accuracy and reduced avascular necrosis rate
Intraoperative Assistance	Batailler et al. (2021)	ROSA robotic knee system	Cadaveric validation study	30 knees	Experimental validation	Bone resection accuracy	Mean alignment deviation ≈0.03° demonstrating high surgical precision
Postoperative Rehabilitation	Rossi et al. (2024)	AI-enabled mobile rehabilitation platform	Prospective cohort study	536 patients (218 active users)	Real-world clinical validation	Rehabilitation adherence	Improved patient engagement and adherence to postoperative rehabilitation
Postoperative Rehabilitation	Miller-Jackson et al. (2022)	Soft robotic hip-flexion exoskeleton with sensor-guided control	Experimental human subject study	10 participants	Controlled laboratory evaluation	Muscle activation (EMG) during assisted hip flexion	Robotic assistance reduced rectus femoris muscle activation by ≈ 40–43%, demonstrating effective rehabilitation support

### 4.1 Diagnosis and imaging

In orthopedic surgery, early applications of AI were predominantly focused on knee and hip interventions, leveraging data from preoperative, intraoperative, and postoperative stages. Its integration into shoulder surgery is a more recent development, with an increasing number of case reports but relatively few large-scale, systematic investigations (Lee et al., 2024). AI algorithms are now being applied to the diagnosis of diverse musculoskeletal conditions, including osteoporosis, fractures, bone tumors, and other orthopedic disorders (Itchhaporia, 2022). Across multiple studies, AI-based diagnostic models have demonstrated comparable or modestly higher accuracy than general practitioners in selected diagnostic tasks and achieved visual recognition performance comparable to that of experienced specialists (Olczak et al., 2017; Myers et al., 2020; Vaishya et al., 2020).

Conventional interpretation of orthopedic imaging is highly reliant on physician expertise and manual annotation of anatomical structures, a process that is both labor-intensive and prone to bias (Bu et al., 2024; Liawrungrueang et al., 2024). Recent advances in AI have transformed fracture risk prediction, particularly in elderly patients with osteoporosis (Hu et al., 2021). Landmark work by previous research highlighted the capacity of CNNs to outperform conventional diagnostic approaches in identifying fracture susceptibility (Tjardes et al., 2020). Architectures such as Double U-Net deliver high-precision segmentation of orthopedic structures and complex lesion delineation (Kim-Wang et al., 2023; Zhang J. et al., 2023). Similarly, GAN-based frameworks enable cross-modal image synthesis and feature alignment, addressing challenges such as indistinct orthopedic margins. These models also support data augmentation in small-sample settings and improve annotation efficiency, helping to mitigate limitations posed by small datasets. Additionally, adversarial training enhances segmentation robustness and adaptability to noise and imaging artifacts (Kessler et al., 2020; Nishiyama et al., 2021). Collectively, these developments have advanced automated segmentation to the point where AI-driven systems can detect fractures, degenerative joint disease, and soft tissue injuries more rapidly, and in many cases more accurately, than traditional methods (Vaishya et al., 2025).

AI has also advanced quantitative risk assessment. Machine learning algorithms that integrate clinical data—such as the FRAX model—have been widely applied for quantitative assessment of fracture risk, combining bone mineral density with multidimensional clinical parameters (Compston, 2015). Building on these approaches, recent AI-based models aim to enhance individualized risk stratification by incorporating heterogeneous datasets, including radiographic features, clinical history, and patient demographics, thereby supporting earlier identification of patients at high risk of fracture (Wu et al., 2023).

Image classification represents another important advancement in diagnostic precision and treatment planning. Accurate grading and staging of orthopedic diseases are important to guiding appropriate therapeutic strategies, as different grades often require distinct interventions; errors in grading can therefore have serious clinical consequences (Zech et al., 2022). Traditionally, tasks such as lesion localization demand substantial specialist expertise, and complex tumors or fractures may still be misclassified (Han et al., 2025).

Deep learning-based models such as Faster R-CNN and YOLO can rapidly process medical images to localize pathological lesions with high precision (Li et al., 2023a; Liao et al., 2023). CNN-based classifiers extend this by embedding large volumes radiographic data, clinical expertise, and patients’ clinical information, thereby improving diagnostic accuracy while reducing clinician workload, processing time, and lowering misdiagnosis rates (Zhou et al., 2022; Lee S. H. et al., 2023). For instance, Kalmet applied CNN-based models to correlate osteoarthritis severity with structural degeneration, enabling patient stratification based on simulated bone structure (Kalmet et al., 2020). Similarly, automated grading of lumbar disc lesions on MRI has achieved detection accuracy of 95.6% (Inoue et al., 2022).

In bone tumor diagnostics, AI-powered deep learning models have been shown to not only differentiate malignant from benign lesions but also predict patterns of tumor invasion, offering valuable information for surgical planning (Meng et al., 2023). In trauma orthopedics, AI serves as a critical tool for the comprehensive evaluation of chronic osteomyelitis, where advanced algorithms delineate infected bone regions, quantify bacterial load within the medullary cavity, assess vascular status, and generate safety classifications that guide subsequent management strategies (Shi et al., 2023). Computer-aided systems employing CNNs have also been developed for bleeding site detection, enhancing the identification of vascular injuries associated with fractures and enabling faster diagnosis and timely intervention in acute trauma care (Huang et al., 2019; Soyer et al., 2022). AI-based approaches have also advanced fracture classification, with novel deep learning algorithms achieving accurate categorization across multiple imaging modalities, including radiography, CT, and MRI (Park et al., 2022; Safran et al., 2023).

**FIGURE 4:**
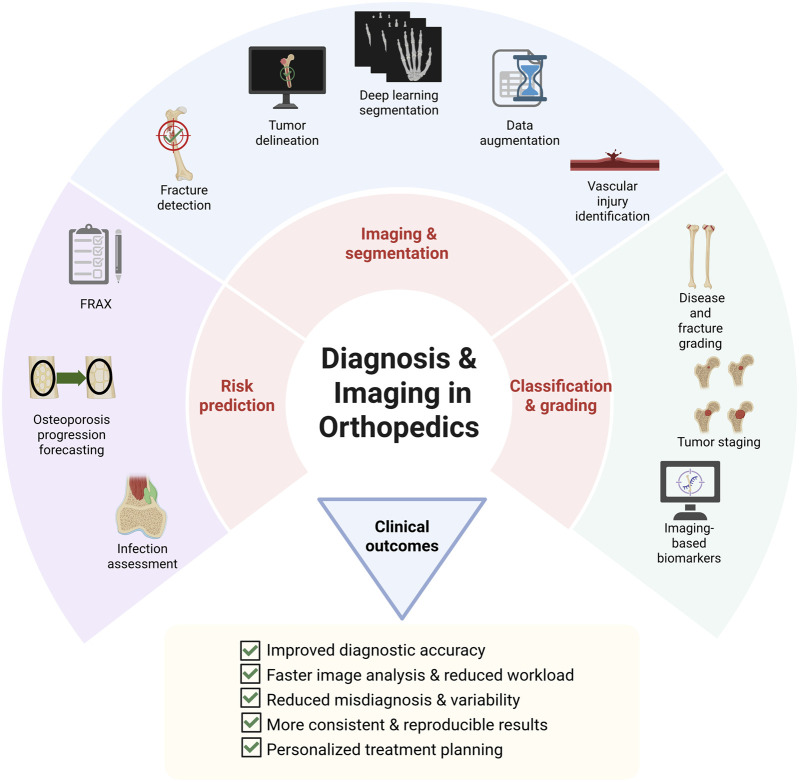
Conceptual overview of AI applications in orthopedic diagnosis and imaging, spanning risk prediction, imaging and segmentation, classification and grading, with outcomes including improved accuracy, reduced workload, and personalized treatment planning. This figure is a conceptual schematic drawn based on evidence and references discussed in Section 4.1.

Despite rapid progress, several challenges continue to limit the clinical translation of AI in orthopedic imaging. Selecting and developing optimal models, ensuring effective integration with heterogeneous clinical datasets, and reducing algorithmic bias remain central obstacles (Zhang et al., 2022). Most current systems range from advanced research prototypes to early-stage clinical deployment, with external validation often limited to retrospective, single-center datasets. Equally important are interpretability and reproducibility, which are critical to fostering surgeon confidence and safeguarding patient safety, particularly as models may be susceptible to performance drift when applied across institutions or imaging platforms. Even so, ongoing advances are steadily broadening the role of AI—from fracture detection, fracture risk prediction, and disease grading, where evidence is strongest, to more complex diagnostic decision-making workflows, where evidence remains comparatively limited. With continued refinement and rigorous validation, these technologies are poised to reshape orthopedic diagnostics and significantly improve patient outcomes. These applications are summarized in Figure 4.

### 4.2 Preoperative planning

AI holds substantial potential to advance orthopedics, a specialty characterized by a high volume of surgical procedures, through its integration into preoperative planning. Beyond conventional image classification, AI-driven three-dimensional (3D) modeling enables precise, patient-specific anatomical reconstructions from CT or MRI data, offering surgeons more intuitive and comprehensive visualization of anatomical regions (Marsilio et al., 2023). Advanced architectures such as the Enhanced Attention Res-UNet, which integrates residual networks with U-Net structures and attention mechanisms, have markedly improved bone segmentation accuracy and complex feature extraction, thereby supporting the creation of high-fidelity 3D models for surgical planning (Marsilio et al., 2023). Similarly, 3D U-Net model offers exceptional precision in delineating bony structures, enhancing prosthesis matching, and supporting surgical navigation, which collectively improve the efficiency and accuracy of orthopedic interventions (Gillot et al., 2022; Yan et al., 2022). These innovations overcome the limitations of traditional 3D modeling by capturing fine anatomical variations and providing surgeons with comprehensive views of pathological regions, ultimately enabling more precise operative strategies (Zhang H. et al., 2023; Wei et al., 2025; Al-Smadi et al., 2026). For example, in spinal deformity correction, AI-based 3D modeling can quantify vertebral rotation and curvature deviations with errors reduced to within 0.5°, thereby refining surgical pathway design and improving both operative outcomes and surgeon-patient communication (Zhang H. et al., 2023).

Selecting the optimal surgical procedure is a pivotal step in orthopedic care, as it directly influences treatment effectiveness, complication rates, and overall patient quality of life (Rompianesi et al., 2022). Traditionally, this decision relies on the surgeon’s expertise and clinical judgment; however, AI models are increasingly being employed to support surgical planning and procedure selection. By integrating patient-specific clinical data with advanced imaging analytics, these systems generate individualized surgical strategies, predict postoperative outcomes, and streamline workflows, thereby reducing decision-making time and minimizing unnecessary surgical trauma (Trasolini and Byrne, 2021; Henderson et al., 2024; Lin et al., 2024). Beyond planning, AI algorithms trained on large datasets can also estimate key perioperative parameters, including mortality risk, likelihood of transfusion, and projected hospital length of stay following elective arthroplasty (Harris et al., 2019; Jo et al., 2020; Wei et al., 2021; Innocenti et al., 2022).

A growing number of AI-based predictive models have been developed to integrate large volumes of patient data with the aim of improving clinical outcomes. For instance, a study involving 28,742 patients from the U.S. National Surgical Quality Improvement Program compared a conventional machine learning-based ANNs model with traditional logistic regression for predicting safe same-day discharge following TKA, demonstrating comparable accuracy in identifying key clinical determinants (Wei et al., 2021). Extending these applications, another study reported that a deep learning algorithm outperformed the American Society of Anesthesiologists’ conventional scoring system in estimating mortality and morbidity risk after spinal fusion (Kim et al., 2018; McDonnell et al., 2021). Similar findings were observed in a large-scale analysis of 111,147 patients undergoing primary shoulder arthroplasty, where ANN-based models achieved 73.1%–91.8% accuracy in forecasting hospital costs, length of stay, and discharge disposition across both degenerative and traumatic conditions (Karnuta et al., 2020).

**FIGURE 5:**
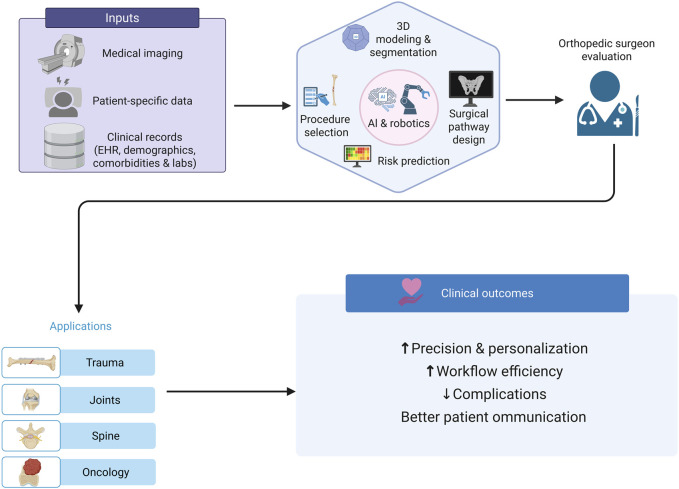
Conceptual schematic illustrating AI-supported preoperative planning in orthopedic surgery: integrating patient-specific data, imaging, and risk prediction with 3D modeling and surgical pathway design to enhance precision, reduce complications, and improve workflow efficiency across trauma, joint, spine, and oncology applications. This figure is a conceptual schematic drawn based on evidence and references discussed in Section 4.2.

Beyond decision support, AI contributes to surgical pathway optimization. Conventionally, trajectory selection has depended largely on a surgeon’s expertise. In contrast, AI-based systems can autonomously optimize and refine surgical trajectories, subsequently assisting surgeons in enhancing precision and supporting real-time decision-making (Kalanjiyam et al., 2024). This capability is especially valuable in bone tumors, where the integration of AI with robotic platforms facilitates the design of accurate surgical strategies, streamlines workflows, and reduces procedural complexity (Sung and Poon, 2020; Suri et al., 2024). For instance, an AI-based approach for preoperative evaluation of bone tumors demonstrated superior sensitivity in detecting changes in tumor size and lymphatic invasion compared with conventional imaging modalities such as MRI and CT (Tjardes et al., 2020). This enhanced diagnostic capability enables more precise surgical planning and contributes to improved clinical outcomes. An overview of AI-driven preoperative planning and its clinical applications is presented in Figure 5.

The selection of appropriate surgical procedures is critical for optimizing patient outcomes, reducing complication rates, and enhancing overall quality of life (Jayakumar et al., 2021; Chen, 2023). At present, most AI-driven preoperative planning tools function as clinician decision-support systems, with selective clinical deployment primarily in high-volume arthroplasty and spine centers, while broader adoption remains limited. External validation is variable, and many studies rely on retrospective, single-center imaging datasets, which constrains generalizability across institutions and implant systems. Key challenges include data fragmentation, potential algorithmic bias, limited transparency of AI-generated recommendations, and uncertainty regarding clinical accountability. Even so, ongoing innovation in AI-driven technologies offers the potential to improve implant templating, alignment optimization, and trajectory planning—where evidence is currently strongest—while more comprehensive, fully automated surgical pathway selection remains comparatively less mature. With continued refinement, secure data-sharing frameworks, and prospective multicenter validation, these technologies may further improve the accuracy, generalizability, and clinical applicability of AI in surgical planning and personalized orthopedic care.

### 4.3 Intraoperative assistance

Orthopedic diseases require timely intervention following accurate diagnosis, with surgery often representing the definitive treatment approach (Li et al., 2023b). In many cases, surgeons are frequently required to make rapid, irreversible decisions under highly individualized patient conditions, relying on their expertise and real-time judgment—a process that is inherently vulnerable to variability and bias (Han et al., 2025). The integration of AI into orthopedic surgery, especially CNNs, has shown considerable promise for intraoperative applications, including robotic control, real-time data processing, and adaptive adjustment of surgical strategies (Han et al., 2025). By supporting surgeons in navigating complex surgeries, AI improves procedural safety and precision. Furthermore, as surgical robotics become increasingly common in orthopedic practice, AI plays a pivotal role in enabling more efficient, faster, and safer operations, ultimately enhancing patient outcomes (Maffulli et al., 2020; Jeyaraman et al., 2023; Thomas, 2023).

Accurate intraoperative localization remains a major challenge in orthopedic surgery, particularly in cases involving fractures embedded within soft tissues or multiple bone fragments. These scenarios complicate image-assisted guidance, prolong operative duration, and increase procedural risks for both patients and surgeons (Wang M. et al., 2024). To overcome these challenges, AI-based technologies have been introduced to enhance localization of not only fracture sites but also bone tumors. For instance, one study employed deep learning to develop a system for intraoperative tumor mapping in osteosarcoma, thereby enabling more precise surgical decision-making and improving operative accuracy (Guan et al., 2025).

A major advancement lies in the integration of AI with multimodal imaging and robotic platforms. At this stage, AI plays a critical role in enhancing image fusion and anatomical recognition. By combining intraoperative imaging modalities such as X-ray, CT, MRI, and ultrasound with preoperative three-dimensional models, AI systems can generate continuously updated anatomical reconstructions, providing surgeons with high quality real-time navigation to inform decision-making (Cotic et al., 2024; Goodman et al., 2024). For instance, in spinal surgery, robotic navigation platforms have been shown to integrate intraoperative imaging with preoperative plans to produce dynamic anatomical maps that improve surgical accuracy (Wang T. Y. et al., 2021). Building on these advances, AI-driven robotic systems incorporating deep learning such as the TiRobot platform and the ROSA surgical assist system have further refined intraoperative implant placement, achieving higher levels of precision and reliability (Fiske et al., 2019).

**FIGURE 6:**
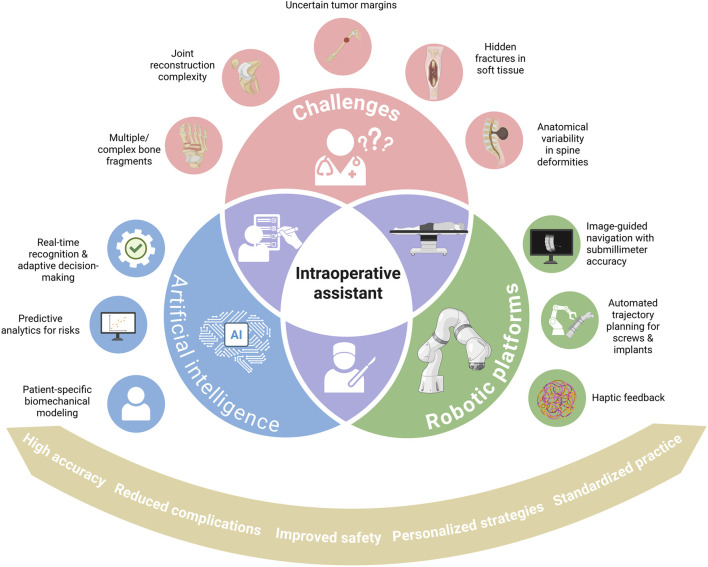
Conceptual illustration depicting the role of AI in intraoperative imaging integration, navigation, and robotic-assisted execution under surgeon control. This figure is a conceptual schematic drawn based on evidence and references discussed in Section 4.3.

The TiRobot system integrates advanced optical tracking with robotic arm technology, achieving sub-millimeter positioning accuracy (Huang et al., 2021). This level of precision is particularly valuable in complex orthopedic procedures, where it can substantially improve both surgical accuracy and patient safety (Yang and Seon, 2023). Similarly, the ROSA surgical assist platform provides anatomical measurements at preoperative, intraoperative, and postoperative stages. Its fixed-arm design maintains stable instrument orientation during implantation, while clinical studies of pedicle screw placement have demonstrated reductions in postoperative bleeding, complication rates, and operative time (Batailler et al., 2021; Liu J. et al., 2021). Together, these platforms illustrate how AI-integrated robotic systems may advance intraoperative accuracy and patient safety by standardizing surgical execution and minimizing procedure-related risks. An overview of AI and robotic platforms as intraoperative assistants, their challenges, and clinical benefits is presented in Figure 6.

The convergence of robotic technologies and AI is progressively transforming orthopedic surgery by enhancing preoperative planning, improving intraoperative precision, and optimizing postoperative outcomes across a variety of procedures, including those involving the knee, hip, and shoulder. At present, robotic-assisted systems represent the most clinically mature application of AI integration in orthopedics, with routine deployment for navigation and instrumentation in arthroplasty and spinal surgery, while other applications remain more limited in scope. These advancements enable surgeons to achieve greater accuracy in fracture reduction, implant positioning, and anatomical alignment, supported by evidence derived largely from platform-specific and single-center cohorts, with external multicenter validation still evolving. Moreover, AI offers the capability to individualize surgical strategies by incorporating patient-specific factors such as anatomical variation and medical history; however, key risks include limited interpretability of AI-generated guidance, interoperability challenges, performance drift following software updates, and safety concerns related to registration or hardware failure. Overall, evidence is strongest for navigation accuracy and implant positioning, whereas autonomous intraoperative decision-making and task execution remain comparatively immature and investigational.

### 4.4 Postoperative care and rehabilitation

AI is also reshaping postoperative care and rehabilitation in orthopedics. Effective management of postoperative complications is critical to orthopedic patient recovery, yet conventional approaches often depend heavily on physician expertise, which can be time-consuming and lack personalization. By integrating multimodal datasets, AI enables automated analysis, risk prediction, remote patient monitoring, and decision support, thereby improving the efficiency of postoperative workflows, enabling earlier complication detection, and facilitating individualized nursing strategies (Hornung et al., 2022; Digumarthi et al., 2024; Prudnikov et al., 2024; Wanees et al., 2024). Large-scale models trained on patient registries have shown promise in predicting postoperative complications across diverse orthopedic populations (van Boekel et al., 2024; van der Meijden et al., 2024).

Beyond complication monitoring, AI is being leveraged to optimize postoperative pharmacotherapy. Fracture healing is a prolonged process, effective drug management is essential to ensure recovery, yet certain medications may interfere with physiological bone repair (Liu P.-R. et al., 2021). To address this challenge, deep learning models have been developed to identify and classify drug-drug interactions by analyzing their components and determining whether they enhance or inhibit each other’s effects (Romm and Tsigelny, 2020). In osteoporosis management, similar algorithms have been applied to predict postoperative drug interactions, allowing clinicians to optimize dosing regimens, lower long-term healthcare costs, and improve patient adherence (Konradi et al., 2022).

The postoperative period has become a central focus of AI-driven innovation, particularly in the realm of personalized rehabilitation. Conventional rehabilitation programs are often limited by poor individualization and inconsistent patient adherence, challenges that AI can address through dynamic data analysis, real-time exercise supervision, and tailored feedback (Alsareii et al., 2022; Han et al., 2025). Increasingly, smartphone-based platforms have been employed to continuously monitor rehabilitation progress and vital signs following TKA (Myers et al., 2020; Kurmis and Ianunzio, 2022). Machine learning-based systems can monitor physiotherapy participation, track exercise adherence, and notify healthcare providers when recovery milestones are not achieved (Batailler et al., 2022).

Recent advancements in intelligent rehabilitation systems have further demonstrated the potential of AI-assisted technologies to support complex motor recovery (Song et al., 2025). Averta et al. developed a human-like motion generation algorithm based on functional principal component analysis to analyze and synthesize complex human movements, including free motion and obstacle avoidance, providing insights into motion modeling for rehabilitation applications (Averta et al., 2020). Building on human motion analysis principles, Zhao et al. designed a tele-rehabilitation system integrating natural human responses with big-data analytics, incorporating an upper-limb rehabilitation robot that combines flexible ropes and exoskeleton components to assist clinicians in optimizing individualized rehabilitation programs (Zhao et al., 2020). Extending robotic applications to lower-extremity rehabilitation, Miller-Jackson et al. developed a soft pneumatic actuator-driven exoskeleton for hip flexor rehabilitation, demonstrating significant reductions in muscle activation during assisted leg lifting, suggesting potential benefits for individuals with mobility impairments (Miller-Jackson et al., 2022). In parallel with hardware-based solutions, digital platforms are increasingly being used to support rehabilitation management. For example, Rossi et al. investigated the use of the smartphone-based care management platform myMobility for postoperative rehabilitation following total knee arthroplasty, highlighting the potential of mobile technologies to improve patient engagement and rehabilitation adherence (Rossi et al., 2024).

The integration of AI with augmented reality (AR) and virtual reality (VR) technologies has introduced a new paradigm in postoperative orthopedic rehabilitation. Advances in wearable multidimensional motion sensors now allow for precise detection of subtle movement patterns while capturing real-time biomechanical data across multiple body regions, thereby expanding the possibilities for individualized rehabilitation programs (Wang Q. et al., 2021; Guo et al., 2023; Paul, 2024). Within VR-based training environments, AI can continuously monitor patient performance in real time and dynamically adjust exercise intensity, optimizing therapeutic efficiency while minimizing the risk of overtraining-related injuries (Hu et al., 2023; Youssef et al., 2024).

**FIGURE 7:**
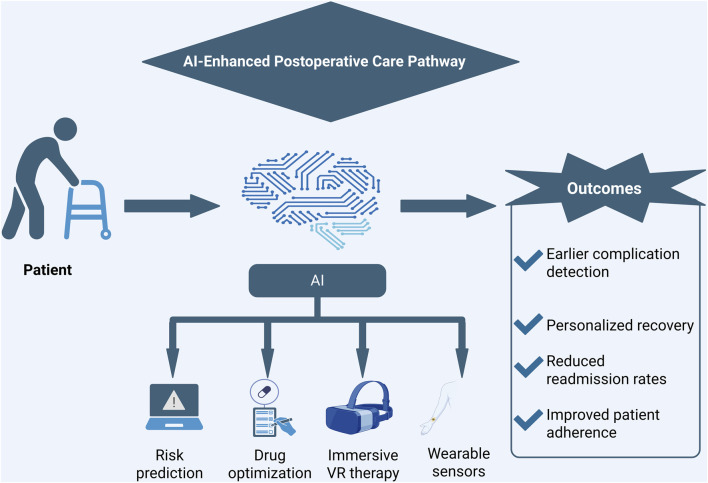
Conceptual schematic summarizing AI-enhanced postoperative care pathway in orthopedics, integrating risk prediction, drug optimization, VR-based rehabilitation, and wearable sensors to enable earlier complication detection, personalized recovery, reduced readmission rates, and improved patient adherence. This figure is a conceptual schematic drawn based on evidence and references discussed in Section 4.4.

Such adaptive systems enhance the consistency, accessibility, and overall effectiveness of orthopedic rehabilitation. A notable example is an immersive VR-based robotic rehabilitation platform has been developed that integrates intelligent walker-assisted mobility, gait parameters, and VR-guided gait training. Using a 3D wearable motion capture system, bilateral lower limb kinematics were recorded alongside cybersickness symptom assessments via patient questionnaires. This AI-enhanced VR system improved neuromuscular control, independence, and locomotor recovery, thereby enabling more precise and personalized rehabilitation protocols (Loureiro et al., 2024). These applications of AI in postoperative care and rehabilitation—spanning risk prediction, drug optimization, immersive VR therapy, and wearable sensors—are summarized in Figure 7.

Taken together, AI is emerging as a powerful tool that unites preoperative, intraoperative, and postoperative data streams to guide clinical decision-making and optimize outcomes. At present, most AI-driven postoperative care and rehabilitation systems remain at the research or early pilot deployment stage, with limited large-scale clinical integration and external multicenter validation. While challenges remain, particularly regarding data standardization, methodological validation, and dataset heterogeneity—often characterized by small, single-center cohorts and short follow-up—key risks include algorithmic bias in activity recognition, performance drift across wearable devices, interpretability limitations, privacy concerns, and safety in unsupervised home-based use. Even so, the integration of AI with robotic assistance, imaging, gait analysis, and immersive VR/AR platforms shows strongest evidence for activity monitoring, adherence assessment, and short-term functional evaluation, whereas fully automated rehabilitation planning and long-term outcome prediction remain comparatively less mature. With continued refinement and prospective validation, these technologies offer a promising path toward more personalized rehabilitation in orthopedic care.

### 4.5 Educational, surgical training and research

AI in orthopedics offers considerable potential to advance both education and research (Masters, 2019; Reid and Eaton, 2019). In surgical training, where repetitive practice and structured curricula are essential for skill acquisition (Guerrero et al., 2023), AI can be integrated with VR and AR platforms to create immersive, procedure-specific simulations (Ghaednia et al., 2021). Leveraging extensive datasets, these simulations can closely replicate real-world operative environments, including complex and atypical scenarios, thereby providing comprehensive, hands-on training opportunities (Roche et al., 2021). Complementary technologies such as 3D printing enable the production of anatomically accurate models for practical exercises, while AI systems can emulate both traditional manipulative techniques and surgical workflows (Han et al., 2019). More recently, AI-enabled emotional simulations have been developed to mimic authentic patient responses, helping trainees cultivate empathy and humanistic care (Guan et al., 2025). This “virtual surgery” paradigm allows surgeons to familiarize themselves with patient-specific anatomy, anticipate potential risk areas, and refine operative strategies—ultimately shortening operative times, improving surgical success rates, reducing intraoperative complications, and lowering healthcare costs (Almarhoumi, 2024; Henderson et al., 2024). The benefits are especially pronounced in technically demanding procedures such as joint arthroplasty (Jang et al., 2024), spinal procedures (Wilson et al., 2025), and bone tumor excision (Rai et al., 2024), where AR and VR are emerging as transformative adjuncts to training.

In orthopedic research, AI is expediting the development of novel biomaterials and treatments. By processing extensive clinical and genomic datasets, AI systems can identify candidate biomarkers for conditions such as osteoarthritis and propose new therapeutic targets (Vaishya et al., 2025). Large language models (LLMs), such as DeepSeek and ChatGPT, have also begun to play a role in research and academic workflows. ChatGPT, for example, has been explored as a supplementary tool for narrative generation in healthcare, though its outputs require careful fact-checking due to risks of inaccuracy (Vaishya et al., 2023). In one investigation assessing ChatGPT’s role in peer review, human evaluations of 21 anonymized manuscripts were compared with those generated by ChatGPT versions 3.5 and 4.0. Although partial concordance was observed—particularly with version 4.0—the study concluded that, despite its potential to enhance efficiency, AI is not yet a substitute for human reviewers (Vaishya et al., 2024). Similarly, DeepSeek, a newly developed LLM, has demonstrated strong performance in medical tasks, including United States Medical Licensing Examination (USMLE) questions and diagnostic reasoning (Tordjman et al., 2025). Nonetheless, persistent challenges—including variability in data quality, the absence of standardized methodologies, and the limited interpretability of AI-generated analyses—continue to constrain clinical adoption. Ethical considerations, algorithmic bias, and the requirement for robust validation further underscore the need for cautious integration of AI tools into orthopedic research.

### 4.6 Data analysis and decision-making

**FIGURE 8:**
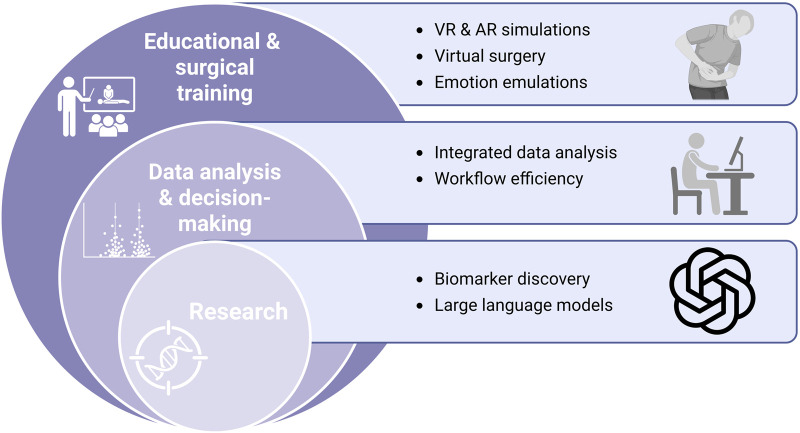
Conceptual schematic summarizing AI applications in orthopedic education, decision-making, and research, encompassing VR/AR-based training, integrated data analysis, workflow efficiency, biomarker discovery, and large language models. This figure is a conceptual schematic drawn based on evidence and references discussed in Sections 4.5, 4.6.

Intelligent orthopedics offers substantial potential to advance shared decision-making, fostering collaboration both between healthcare professionals and between clinicians and patients (Link and Pedoia, 2022). Such an approach can help curb unnecessary use of clinical resources and mitigate tensions in the doctor-patient relationship (Liu P. et al., 2021). A key component of modern orthopedic practice is integrated data analysis, an area in which AI has demonstrated considerable impact. For example, Johnson applied AI to analyze 657 adolescent cases of greenstick fractures, identifying a strong correlation between vascular supply and prognosis, and achieving recovery prediction accuracies exceeding 95% (Johnson et al., 2018). Similarly, Nuance’s medical speech recognition platform that has been shown to enhance workflow efficiency by reducing report-entry time to one-fifth of the conventional duration, thereby enhancing doctor productivity (Katzman et al., 2023). Collectively, these advances highlight AI’s capacity to enhance diagnostic precision, streamline orthopedic workflows, and support evidence-based clinical decision-making. However, effective implementation necessitates seamless integration into existing clinical infrastructures, coupled with rigorous validation to ensure reliability, transparency, and user confidence. Future efforts should prioritize optimizing these systems for wider clinical adoption, while addressing data security, ethical oversight, and governance challenges. The applications of AI across educational training, research, and decision-making are summarized in Figure 8.

Although many studies evaluating artificial intelligence in orthopedic surgery report algorithmic performance metrics such as sensitivity, specificity, and area under the receiver operating characteristic curve (AUC), these indicators primarily reflect technical model performance rather than direct clinical benefit. In this review, the clinical implications of AI are discussed across the application-focused sections of the manuscript. Specifically, Sections 4.1–4.4 describe how AI technologies influence clinical decision-making and patient outcomes, including improved diagnostic accuracy, enhanced surgical planning and risk prediction, increased intraoperative precision through robotic assistance, and improved postoperative monitoring and rehabilitation strategies. Nevertheless, many studies remain limited to retrospective algorithm validation, and future research should prioritize prospective clinical investigations evaluating how AI-assisted systems affect patient outcomes, healthcare efficiency, and surgical decision-making.

## 5 Challenges and unresolved issues

**FIGURE 9:**
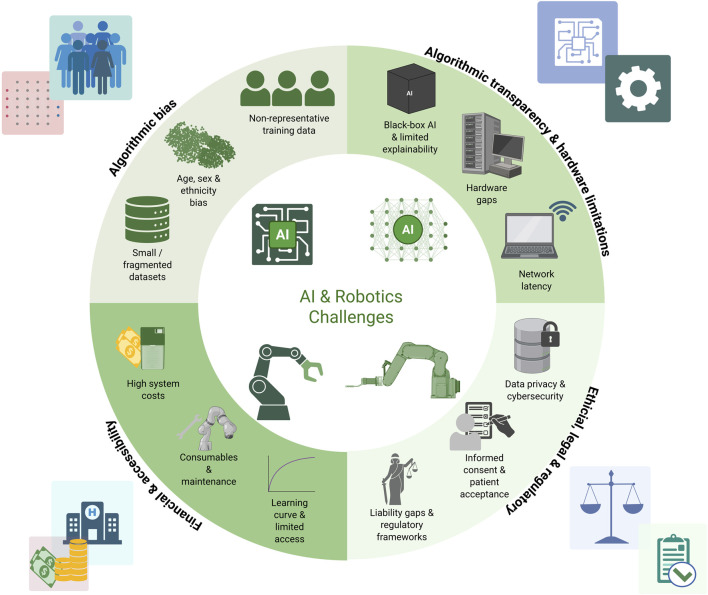
Conceptual synthesis of major challenges in the integration of AI and robotics into orthopedic surgery, spanning data limitations, algorithmic opacity, hardware constraints, interoperability issues, bias, financial barriers, and ethical-legal concerns. This figure is a conceptual schematic drawn based on evidence and references discussed in Section 5.

The key challenges and unresolved issues associated with AI and robotics in orthopedic surgery are summarized in Figure 9.

### 5.1 Algorithmic transparency and hardware limitations

AI algorithms form a robust technical foundation for intelligent assistance in orthopedic surgery, yet their translation into clinical practice remains constrained by key technical limitations. Foremost among these is the “black-box” nature of many AI models, which compromises interpretability and, consequently, adoption and clinical trust (Blackman and Veerapen, 2025; Zheng et al., 2025). In orthopedic surgical settings, doctors need to understand the reasoning behind AI-derived predictions to assess the validity of proposed operative pathways or risk assessments. Yet the opacity of algorithmic decision-making, coupled with the inherent complexity of underlying computational processes, often limits a surgeon’s ability to evaluate the logic of outputs, thereby reducing confidence and hindering real-world application (Kumar et al., 2022; Ramkumar et al., 2022; Pai et al., 2024). In recent years, advances in explainable AI have begun to address this challenge. For example, heatmap-based visualization tools can illustrate the specific anatomical regions or features influencing model outputs, enabling surgeons to better interpret AI recommendations and fostering greater trust in their application (Akula et al., 2022; Mukhtorov et al., 2023).

A central objective of digital orthopedics is to optimize resource utilization; however, current algorithmic frameworks often demand substantial human input for design, validation, and refinement. For instance, in one study aimed at developing an AI system capable of automatically detecting metacarpal fracture lines, the initial model failed to achieve satisfactory accuracy. Consequently, 10 specialist physicians engaged in a 3-month training process to iteratively teach the system, enabling it to acquire basic automated recognition capabilities (Kim et al., 2024).

Beyond algorithms, the hardware platforms that enable AI applications, such as intelligent navigation platforms, surgical robots, and sensor-based devices, are critical in translating computational outputs into precise clinical actions. Their performance and reliability directly determine clinical value, yet several barriers remain. A major challenge lies in the absence of standardized interfaces for heterogeneous data sources, which constrains the efficiency of multimodal data integration. In orthopedic procedures, variations in data formats between electronic health records (EHRs), imaging systems, and biomechanical sensors compel developers to create bespoke data-conversion modules for each device, increasing system complexity, prolonging development cycles, and introducing risks of data transmission errors (Su and Pei, 2024).

Technical limitations in intraoperative adaptability further impede precision. While certain orthopedic robotic systems employ generative AI to construct three-dimensional skeletal models, their compensation algorithms for soft tissue deformation still rely primarily on static preoperative data and lack the capacity to fully adapt to dynamic intraoperative load variations, leading to suboptimal feedback during procedures (Ryu et al., 2024; Zhang Z. et al., 2024). Furthermore, device incompatibility and limited system-level interoperability impede technological evolution. Discrepancies in communication protocols and operating systems among devices hinder real-time data synchronization, complicate intraoperative AI deployment, and disrupt the seamless incorporation of AI into orthopedic workflows (Sherrod et al., 2023). In addition, the predominant dependence on cloud-based computing introduces network latency, which can delay robotic arm responses; in high-precision settings, such delays may heighten the risk of neurovascular injury and undermine the clinical feasibility of AI-assisted interventions (Jiang S.-H. et al., 2024). Looking ahead, future research should prioritize the development of interpretable, real-time AI algorithms with integrated biomechanical feedback, as well as hardware innovations that ensure seamless interoperability and localized computing to eliminate latency risks. Establishing standardized data-sharing frameworks and advancing multimodal AI models capable of addressing complex clinical scenarios will be crucial for unlocking the full potential of AI in orthopedic surgery.

### 5.2 Algorithmic bias

Algorithmic bias remains a significant barrier to the equitable application of AI in orthopedic surgery, particularly when these models are trained on non-representative datasets. Evidence indicates that algorithms developed predominantly using data that disproportionately reflect specific demographic groups, such as defined by sex or race, demonstrate reduced reliability and accuracy when applied to underrepresented populations (Arjomandi Rad et al., 2025; Khan et al., 2025). For example, an evaluation of AI models for osteoporosis classification from chest radiographs demonstrated high susceptibility to selection bias, directly attributable to insufficient diversity in the training data (Yamamoto et al., 2024). Similarly, another study reported that vertebral fracture detection algorithms exhibited diminished accuracy in older patients with multiple comorbidities, underscoring the presence of age-related bias (Namireddy et al., 2024). In sports medicine, differences in bone mineral density and Achilles tendon length have been observed between athletes of variant ethnicities; however, current algorithms fail to account for these variations, perpetuating disparities in diagnosis and treatment (Guan et al., 2025).

Bias is compounded by the fragmented nature of healthcare data, as institutions frequently operate in silos with minimal data exchange, leading to duplicated, non-standardized, and incomplete records (Guan et al., 2025). Overcoming these limitations requires the inclusion of multicenter, demographically diverse datasets in AI development, encompassing a broad spectrum of genders, races, and age groups to enhance model generalizability and clinical applicability (Huffman et al., 2024). In parallel, the establishment of standardized datasets and the refinement of preprocessing techniques for heterogeneous data inputs are needed to reduce sensitivity to population-specific variables and improve algorithmic fairness (Hendricks-Sturrup et al., 2023; Sedano et al., 2025).

### 5.3 Financial and accessibility challenges

AI adoption in orthopedics is also constrained by substantial capital expenditure and ongoing financial burdens for healthcare systems, which may hinder its broader implementation (Han and Tian, 2019; Batailler et al., 2022). The high costs stem not only from the initial investment in hardware but also from recurring expenses for consumables used in each procedure (Christen et al., 2022). In certain regions, these initial and maintenance costs pose significant challenges, particularly for smaller hospitals and private practices that may lack the financial capacity to adopt such technologies (St Mart and Goh, 2021; Kolessar et al., 2022; Rivero-Moreno et al., 2023). The steep learning curve further complicates implementation, as clinical teams must undergo extensive training and adapt to new, technically demanding roles—although this burden may be mitigated when procedures are led by surgeons with prior experience in robotic-assisted techniques (Schopper et al., 2023). Comprehensive cost-benefit analyses are therefore essential to determine whether the integration of robotics and AI in orthopedics yields interventions that are truly cost-effective (Close et al., 2013; Sandhu, 2019; Rajan et al., 2022). Therefore, continued technological innovation, targeted research, and supportive programs, particularly those designed for resource-limited settings, will be critical to ensuring that the benefits of robotic and AI-assisted surgery become accessible and affordable across diverse healthcare systems worldwide.

### 5.4 Ethical, legal and regulatory considerations

In medicine, AI is transforming diagnostics, enhancing surgical precision, and advancing personalized care. Yet alongside these benefits, the integration of AI into orthopedics introduces complex ethical challenges that demand rigorous evaluation and regulatory oversight. Foremost among these is the issue of data privacy. While patient autonomy and informed consent remain foundational to medical practice, AI systems, particularly those based on CNNs and other deep learning architectures, complicates these principles. Such systems require the collection, storage, and analysis of vast datasets, raising substantial concerns about patient confidentiality, data security, and the protection of individual rights (Mohammed et al., 2025).

Historical incidents, such as the 2015 collaboration between the UK National Health Service and DeepMind, later criticized for violating data protection regulations, underscore the urgency of implementing robust privacy protocols (Bicer et al., 2023). Adherence to established legal frameworks, including the Health Insurance Portability and Accountability Act (HIPAA) and the General Data Protection Regulation (GDPR), is critical to maintaining patient trust and ensuring ethical data stewardship (Bicer et al., 2023). In orthopedic surgery, these concerns are compounded by the integration of AI into robotic navigation and intraoperative guidance systems, which depend on continuous data exchange. AI platforms embedded in diagnostic systems and surgical robotics remain vulnerable to cybersecurity threats, including adversarial attacks, software manipulation, or network intrusions that could disrupt real-time navigation, compromise registration accuracy, or alter implant positioning during surgery. To mitigate these threats, AI infrastructures must incorporate robust protections such as multi-factor authentication, end-to-end encryption, and continuous system monitoring (Andriollo et al., 2025).

Beyond privacy and security, patient acceptance represents another key ethical dimension. While surgeons may value AI for its efficiency and precision, patients often perceive it as unreliable or impersonal. In orthopedic surgery, where trust in surgical judgment is paramount, concerns about diminished surgeon-patient interaction or excessive reliance on automation may undermine confidence. Transparent communication is therefore essential to clarify AI’s role as an assistive tool rather than an autonomous decision-maker (Pesapane et al., 2018). Moreover, patients may struggle to assess the risks and uncertainties associated with AI-derived recommendations, particularly when outputs are based on limited or biased datasets. This places greater responsibility on clinicians to communicate not only the content of AI-generated advice but also its limitations, thereby supporting truly informed consent (Kelly et al., 2019; Safdar et al., 2020; Robinson et al., 2025).

The regulation of AI-based medical systems presents a multifaceted challenge that intersects healthcare governance, technological oversight, and data protection. The rapid pace of AI innovation frequently outstrips the development of legal frameworks, with regulatory requirements differing substantially across jurisdictions. To preserve the reliability of AI tools and safeguard patient welfare, regulatory structures must undergo continuous refinement (Secinaro et al., 2021; Al Kuwaiti et al., 2023). The Food and Drug Administration in the United States has implemented a total product lifecycle approach for AI- enabled medical devices, incorporating measures such as good machine learning practices and predetermined change control plans. This dynamic framework is designed to ensure both adaptability and safety across the full lifespan of an AI product (Benjamens et al., 2020). In China, dedicated policies for AI in healthcare are beginning to take shape, and the National Medical Products Administration has already approved several AI-driven medical devices. However, explicit regulatory provisions for adaptive or continuously learning AI systems remain absent (Díaz-Rodríguez et al., 2023).

The integration of AI-assisted decision-making with robotic execution in orthopedic surgery introduces profound ethical and legal complexities, particularly in attributing responsibility when adverse outcomes arise. Among these, accountability represents perhaps the most pressing and unresolved challenge. Conventionally, surgeons have borne full accountability for intraoperative decisions. However, AI-generated recommendations, derived from large-scale data analytics and advanced algorithms, often extend beyond a clinician’s direct expertise, challenging the applicability of established legal doctrines (Braun et al., 2021; Bleher and Braun, 2022). For instance, in orthopedic trauma surgery, AI algorithms assist in planning the optimal trajectory for screw placement in femoral neck fractures. If postoperative imaging reveals screw misplacement leading to complications such as fixation failure or avascular necrosis, it becomes unclear whether the responsibility lies with the surgeon’s intraoperative decision-making, the AI’s preoperative planning recommendation, or potential inaccuracies in the imaging data used for navigation (Jassar et al., 2022). This ambiguity extends to whether responsibility should be assigned to clinicians, AI developers, or healthcare institutions, creating significant barriers to broader AI adoption in surgical practice (Grassini, 2023; Mooghali et al., 2024). To address this challenge, clear institutional and regulatory guidelines are needed to delineate accountability for AI-assisted outcomes, ensuring that innovation advances without compromising patient safety or professional responsibility (Wang C. et al., 2023).

In summary, to address these ethical and regulatory challenges, several practical measures should be considered. Healthcare institutions should establish clear governance frameworks defining accountability among surgeons, institutions, and AI developers. Robust cybersecurity protocols, including encryption and continuous system monitoring, should be implemented for AI-enabled robotic platforms. Transparent communication with patients regarding the role and limitations of AI in surgical decision-making is essential to support informed consent. Additionally, regulatory agencies should require ongoing post-market surveillance and validation of AI systems, particularly those that adapt over time, to ensure patient safety and clinical reliability.

## 6 Future perspectives and research needs

**FIGURE 10:**
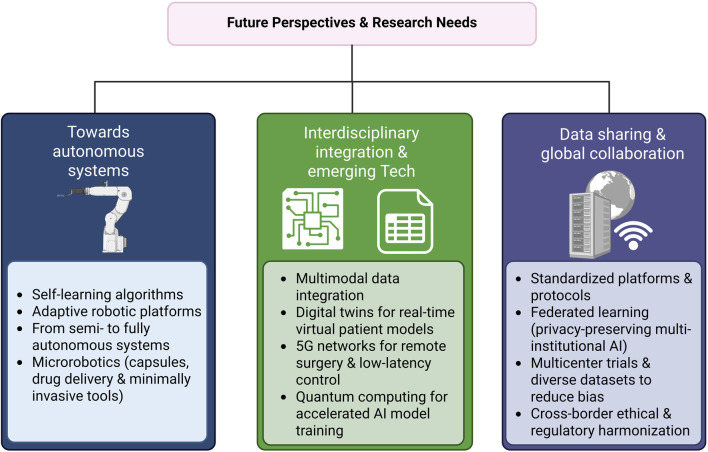
Conceptual overview illustrating future perspectives for AI and robotics in orthopedic surgery, including autonomous systems, interdisciplinary integration with emerging technologies, and global data sharing and collaboration. This figure is a conceptual schematic drawn based on evidence and references discussed in Section 6.

Despite existing challenges and limitations, advances in technology are steadily transforming AI and robotics in orthopedic surgery from a supportive adjunct into an integrated, full-process intelligent system. To facilitate safe clinical translation, future research should prioritize prospective multicenter validation, standardized evaluation frameworks, and integration of AI tools within routine surgical workflows. Looking ahead, development in this field is likely to focus on three key directions: enhancing system autonomy, fostering interdisciplinary integration, and promoting data sharing and global collaboration. These future perspectives and research priorities are summarized in Figure 10.

### 6.1 Towards autonomous systems

Nowadays, AI systems in orthopedic surgery function primarily as assistive technologies, with surgeons retaining ultimate decision-making authority. Advances in self-learning algorithms, coupled with the development of lightweight, intelligent, modular, and highly integrated systems, are driving research toward fully autonomous surgical robotic platforms. These emerging systems integrate adaptive learning capabilities with real-time environmental sensing, enabling them to adjust operative strategies dynamically according to individual patient profiles. Such advancements have the potential to reduce clinical workload and healthcare costs while improving surgical efficiency, consistency, and precision (Marrone et al., 2022; Yi, 2024).

For instance, computer-assisted robotic systems for fracture reduction are beginning to demonstrate semi-autonomous functionalities. Recent progress in this domain shows promise for automating complex orthopedic maneuvers while supporting surgeons intraoperatively (Zhou et al., 2025). In parallel, a study from Johns Hopkins University reported that an AI-driven robotic system enhanced the surgical performance of junior surgeons, achieving performance comparable to that of experienced specialists in specific experimental settings (Romagna et al., 2023). Together, these examples illustrate the accelerating trajectory from assistive toward increasingly autonomous surgical platforms.

The progressive increase in autonomy within robotic surgery also holds the potential to standardize surgical outcomes, reducing variability associated with differences in surgeon experience, training, and daily performance fluctuations (Saeidi et al., 2022). By enhancing procedural reproducibility and providing consistent technical assistance, these systems exemplify the evolving potential of robotics to transform orthopedic surgical workflows.

A particularly promising frontier in this field is micro-robotics, which encompasses the development of portable capsule endoscopes for diagnostic tasks, diverse targeted drug delivery, and minimally invasive surgical procedures (Khandalavala et al., 2020; Probst, 2023). These millimeter-scale microrobots can be navigated using extracorporeal magnetic fields to perform specialized functions. For example, in porcine models, a magnetically guided microrobot equipped with a nitinol clip successfully achieved hemostasis for chronic bleeding during a biopsy (Khandalavala et al., 2020; Probst, 2023). Current research in micro-robotics is concentrated on four primary objectives: developing contained propulsion systems, enhancing miniaturized functionality, ensuring consistent high-quality visualization, and achieving precise telemanipulation capabilities (Khandalavala et al., 2020).

Despite these advances, robotic systems remain tools rather than replacements for the surgeon. Even in highly complex cases that benefit from the enhanced precision of robotic systems, the surgeon’s expertise remains indispensable. As with any surgical instrument, robotic platforms serve as tools, albeit exceptionally sophisticated ones, within the orthopedic surgeon’s armamentarium. For safe clinical adoption, future development of autonomous surgical systems should emphasize human–AI collaboration rather than full automation, ensuring that surgeons retain supervisory oversight of critical decision-making processes. Furthermore, clear regulatory approval pathways, standardized safety evaluation frameworks, and structured surgeon training programs will be essential to support the responsible integration of autonomous robotic technologies into orthopedic practice.

### 6.2 Interdisciplinary integration and emerging technologies

While advances in autonomy demonstrate the growing capabilities of AI-robotic platforms, the next frontier lies in their interdisciplinary integration with emerging technologies. Looking ahead, intelligent orthopedic systems could leverage emerging technologies such as 5G networks to shorten operative times and enable remote surgical control, thereby reducing operator radiation exposure (Guan et al., 2025). In parallel, multimodal data integration holds substantial promise for improving surgical precision. In clinical practice, combining imaging modalities, such as MRI, CT, and ultrasound, with parameters like muscle tension and bone density enables AI platforms to make more comprehensive, patient-specific decisions (Thorn, 2023). For example, integrating three-dimensional CT reconstructions with biomechanical simulations enables the prediction of how different screw diameters and trajectories affect spinal stability, thereby facilitating optimized implant selection. Clinical application of such approaches has already been associated with an 18% reduction in postoperative complications in complex spinal deformity surgeries (Naik et al., 2022).

Compared with traditional computing systems, which are often limited by processing speed and capacity when managing large datasets or complex algorithms, quantum computing offers a substantial advantage. Quantum computing, with its capacity for parallel computation, can accelerate the training of deep learning models, improving the speed and efficiency of biomechanical feature extraction and surgical plan generation (Paul et al., 2024). In parallel, digital twin technology is being explored as a transformative tool, integrating anatomical and physiological data into real-time, continuously updated virtual patient models. These systems allow predictive modeling of surgical outcomes and optimization of treatment strategies, offering considerable potential to improve prognosis and enhance treatment precision (Oeding et al., 2024; Zsidai et al., 2024).

The integration of 5G, digital twin, and quantum computing technologies into clinical orthopedic practice presents several ethical and technical challenges. These include the requirement for large, high-quality datasets; the complexity of system interoperability; and concerns related to data privacy, cybersecurity, and legal accountability. Overcoming these barriers will be essential to achieving seamless, secure, and clinically meaningful implementation. If addressed effectively, these innovations could advance orthopedic surgery toward safer, more precise, and genuinely personalized care.

From a practical perspective, successful deployment will require interoperability with hospital information systems, standardized imaging data formats, and scalable computational infrastructure. Healthcare institutions adopting AI-enabled orthopedic platforms should therefore invest not only in technological infrastructure but also in structured clinical training and multidisciplinary collaboration among surgeons, engineers, and data scientists to support safe and effective clinical integration.

### 6.3 Data sharing and global collaboration

Establishing standardized data platforms and multinational collaborative networks is critical to improving the generalizability of AI models and expanding access to high-quality orthopedic care (Rieke et al., 2020). Achieving this goal requires substantial investment in multi-center large-scale clinical trials to validate AI algorithms across diverse clinical environments and populations, thereby ensuring robust and reproducible performance. Research indicates that integrating data from varied demographic groups, spanning different age, sexes, and races categories, can mitigate algorithmic bias and improve predictive accuracy (Aouedi et al., 2022; Wen et al., 2023). Yet, persistent challenges remain, particularly in navigating data privacy regulations and ensuring adherence to ethical standards, both of which continue to pose significant barriers to global data sharing.

Efforts should focus on enhancing algorithmic transparency and establishing standardized data protocols to enable seamless integration into surgical workflows. Federated learning offers a promising solution, allowing institutions to collaboratively train AI models without exchanging raw data, thereby maintaining both privacy and utility (Li et al., 2019). This approach has already been successfully applied to integrate datasets from 20 international medical centers, enabling the prediction of intensive care unit (ICU) admissions, oxygen requirements, and mortality risks in COVID-19 patients—substantially enhancing model generalizability and accuracy (Dayan et al., 2021).

Looking ahead, the deep integration of AI with AI-enabled imaging, 3D modeling technologies, smart sensors, wearable devices, and robot-assisted systems has the potential to enable real-time patient monitoring and extend personalized care (Mah, 2023; Youssef et al., 2024). Achieving this vision will depend on close collaboration between orthopedic surgeons, data scientists, and healthcare policymakers to address ethical challenges, improve clinical efficacy, and establish regulatory frameworks that ensure the effective and safe adoption of AI-driven innovations in orthopedics (Bekbolatova et al., 2024). To support global collaboration, future initiatives should prioritize the development of international orthopedic data consortia, standardized data governance frameworks, and secure federated learning infrastructures, enabling collaborative algorithm development while maintaining patient privacy and regulatory compliance.

## 7 Limitations

This narrative review has several limitations. First, as a narrative review, it did not follow a formal systematic review protocol or PRISMA guidelines, and a quantitative synthesis or meta-analysis was not performed. Therefore, the selection of studies may be subject to publication and selection bias.

Second, although multiple databases and key literature sources were consulted, the review may not capture all relevant studies, particularly non-English publications or unpublished data. Third, the included studies vary in methodological quality, study design, and clinical validation, and no formal risk-of-bias assessment was conducted.

Finally, the field of artificial intelligence and robotic-assisted orthopedic surgery is rapidly evolving, and some technologies and evidence discussed in this review may change as new research emerges. Despite these limitations, this review provides an up-to-date overview of current applications, challenges, and future directions of AI and robotic technologies in orthopedic surgery.

## 8 Conclusion

The integration of robotic technologies and AI represents a paradigm shift in orthopedic surgery, where the convergence of artificial intelligence and robotics is not merely augmenting existing surgical techniques but fundamentally redefining the standards of precision, safety, and personalization in musculoskeletal care. As evidenced by current applications across diagnosis, surgical planning, intraoperative navigation, and postoperative rehabilitation, these technologies offer tangible benefits in improving surgical accuracy, minimizing complications, and enabling data-driven, patient-specific decision-making.

However, realizing the full potential of AI and robotics in orthopedics demands confronting significant unresolved challenges. These include the lack of algorithmic transparency, persistent biases within datasets, fragmented healthcare infrastructures, and the absence of unified regulatory frameworks capable of overseeing continuously learning systems. Without rigorous validation through large-scale, prospective, and multicenter trials, and without addressing these systemic barriers, the adoption of such technologies risks entrenching disparities rather than advancing equitable care.

Looking ahead, the trajectory of orthopedic innovation is poised to align with the broader digital health agenda: fostering interdisciplinary collaboration, integrating multimodal data through secure and federated platforms, and advancing towards semi-autonomous or autonomous systems that augment rather than replace surgical expertise. Moreover, the integration of AI with emerging technologies—such as digital twins, 5G-enabled remote surgery, and real-time biomechanical modeling—heralds a new era of precision orthopedics that extends beyond the operating theater to encompass lifelong patient monitoring and predictive analytics. To translate these advances into routine clinical practice, future efforts should prioritize prospective multicenter clinical validation, standardized evaluation frameworks for AI-driven surgical systems, and improved interoperability between AI platforms and hospital information infrastructures. In parallel, regulatory bodies and healthcare institutions must develop clear governance frameworks, training programs for surgeons, and secure data-sharing mechanisms to ensure the safe, ethical, and equitable deployment of AI-enabled orthopedic technologies.
